# Developing a best-practice agenda for music therapy research to support informal carers of terminally ill patients pre- and post-death bereavement: a world café approach

**DOI:** 10.1186/s12904-024-01369-8

**Published:** 2024-02-07

**Authors:** Tracey McConnell, Kathryn Gillespie, Noah Potvin, Audrey Roulston, Jenny Kirkwood, Daniel Thomas, Angela McCullagh, Lorna Roche, Marcella O’Sullivan, Kate Binnie, Amy Clements-Cortés, Lauren DiMaio, Zara Thompson, Giorgos Tsiris, Ranka Radulovic, Lisa Graham-Wisener

**Affiliations:** 1 School of Nursing and Midwifery, Queen’s University Belfast, Belfast, UK; 2 Centre for Improving Health-Related Quality of Life, School of Psychology, Queen’s University Belfast, Belfast, UK; 3 Mary Pappert School of Music, Duquesne University, Pittsburgh, PA USA; 4 School of Social Sciences, Education & Social Work, Queen’s University Belfast, Belfast, UK; 5 Independent Researcher, Belfast, UK; 6 CHROMA Therapies, Overross House, Ross Park, Ross on Wye, Herefordshire, UK; 7 MusiCARER Project Carer Advisory Group, Belfast, UK; 8 Wolfson Palliative Care Research Centre, Hull York Medical School, University of Hull, Kingston upon Hull, Yorkshire, UK; 9 Faculty of Music, University of Toronto, Toronto, Canada; 10 Music Therapy, Texas Woman’s University, Denton, USA; 11 Creative Arts and Music Therapy Research Unit, Faculty of Fine Arts and Music, The University of Melbourne, Melbourne, Australia; 12 Division of Occupational Therapy and Arts Therapies, School of Health Sciences, Queen Margaret University, Edinburgh, UK; 13 Clinic for Psychiatry, University Clinical Centre of Serbia, Belgrade, Serbia

**Keywords:** Best practice, Agenda, Music therapy, Informal carers, Pre-bereavement, Bereavement, End-of-life care

## Abstract

**Background:**

Informal carers of terminally ill patients play a vital role in providing palliative care at home, which impacts on their pre- and post-death bereavement experience and presents an up to 50% greater risk for mental-health problems. However, developing and implementing effective bereavement support remains challenging. There is a need to build the evidence base for music therapy as a potentially promising bereavement support for this vulnerable population. This study aimed to co-design an international best practice agenda for research into music therapy for informal carers of patients pre- and post-death bereavement.

**Methods:**

Online half day workshop using a World Café approach; an innovative method for harnessing group intelligence within a group of international expert stakeholders (music therapy clinicians and academics with experience of music therapy with informal carers at end-of-life). Demographics, experience, key priorities and methodological challenges were gathered during a pre-workshop survey to inform workshop discussions. The online workshop involved four rounds of rotating, 25-minute, small group parallel discussions using Padlet. One final large group discussion involved a consensus building activity. All data were analysed thematically to identify patterns to inform priorities and recommendations.

**Results:**

Twenty-two consented and completed the pre-event survey (response rate 44%), from countries representing 10 different time zones. Sixteen participated in the workshop and developed the following best practice agenda. The effectiveness of music therapy in supporting informal carers across the bereavement continuum should be prioritised. This should be done using a mixed methods design to draw on the strengths of different methodological approaches to building the evidence base. It should involve service users throughout and should use a core outcome set to guide the choice of clinically important bereavement outcome measures in efficacy/effectiveness research.

**Conclusions:**

Findings should inform future pre- and post-death bereavement support research for informal caregivers of terminally ill patients. This is an important step in building the evidence base for commissioners and service providers on how to incorporate more innovative approaches in palliative care bereavement services.

**Supplementary Information:**

The online version contains supplementary material available at 10.1186/s12904-024-01369-8.

## Background

The number of people with terminal, life-limiting illness requiring palliative care is increasing at an unprecedented rate as the post-World War II generation ages [1, 2]. International palliative care policy has shifted the focus of palliative care provision from the hospital-based setting into the community [3]. This in turn has increased the number of informal carers worldwide (those who provide unpaid care to close persons) who play a crucial role in providing palliative care at home [4], with international statistics suggesting that informal carers undertake a large proportion of palliative care within the community setting (75–90%) [5]. Despite a large body of evidence showing the negative impact of informal caregiving on physical and psychological wellbeing, there is a dearth of evidence on caregiver bereavement outcomes or best practice interventions for supporting informal carers through grief and bereavement [6].

The death of a loved one, while a common human experience, can be painful [7] characterised by poignant experiences of anger, disbelief and extreme sadness often mixed with positive memories of the deceased [8]. However, most bereaved people can adjust to life without the deceased and do not require professional support [9–11]. Furthermore, bereavement can be an opportunity for personal growth emerging from the healing process.

However, some bereaved people experience a bereavement-related disorder, commonly known as abnormal, pathological, or complicated grief [12]. In 2018, a new diagnostic category – Prolonged Grief Disorder (PGD) - was introduced into the International Classification of Diseases ICD-11 [12] and in March 2022, the American Psychiatric Association (APA) added PGD to the DSM-5-TR [13]. PGD diagnostic criteria for DMS-5 and ICD-11 include persistent yearning for the deceased or feeling pre-occupied with the circumstances of the death [8], and intense emotions that are debilitating or cause extreme distress [14]. However, one important difference is that the ICD-11 permits a diagnosis to be made by clinical assessment after six months [15, 16], whereas the DSM-5 criteria stipulates that a diagnosis of PGD cannot be made until 12 months after the death [8]. The subset of those bereaved who develop PGD (6–10%) [17] experience long-term negative impacts on their psychological, physical and social functioning [11, 16, 18].

Informal carers of those with life-limiting illness often experience anticipatory grief, which can be understood as a pre-death-bereavement phase where the informal carer grieves for losses associated with their loved one’s deterioration in health for some time prior to their death [18, 19]. For example, informal carers of people living with conditions with protracted disease trajectories observe their loved one’s physical, cognitive, social, and psychological deterioration while simultaneously grieving the loss of their pre-caregiver identity as a result of their increased caregiver role [20]. Given the potential for these negative impacts, it is imperative to holistically assess and review the needs of informal carers providing palliative care across the illness trajectory into bereavement.

As there is no agreed time in the literature for when the pre-death-bereavement period begins, the National Institute for Health and Care Excellence (NICE) Guidance for End-of-Life Care for Adults in the United Kingdom [21] is used as a guide to which populations may require pre-bereavement support. For example, this guideline includes conditions such as dementia with an unpredictable prognosis and disease trajectory, where people living with the condition may reach end-of-life prior to the advanced stages of disease [22].

People are deemed to be approaching the end of life when they are likely to die within the next 6–12 months, although this is not always possible to predict. This includes people whose death is imminent, as well as people who:

- have an advanced incurable illness, such as cancer, dementia or motor neurone disease
- are generally frail and have co-existing conditions that mean they are expected to die within 12 months
- have existing conditions if they are at risk of dying from a sudden crisis in their condition
- have a life-threatening acute condition caused by a sudden catastrophic event, such as an accident, stroke or medical complications [21]

The research evidence-base on how to support informal carers across the illness trajectory and into bereavement is under-developed [23]. The most commonly evaluated interventions include bereavement social groups and psychological and counselling interventions. There is however emergent evidence in arts-based approaches [23] A recent World Health Organization (WHO) evidence synthesis [24] highlighted how the arts can improve health and wellbeing in persons with complex medical and psychological issues where current solutions are not optimal.

### Music therapy

“Music therapy is the professional use of music and its elements as an intervention in healthcare, educational, community and everyday environments with individuals, groups, families, or communities who seek to optimise their quality of life and improve their physical, social, communicative, emotional, and spiritual health and wellbeing. Research, practice, education, and clinical training in music therapy are based on professional standards according to cultural, social, and political contexts” [22]. Music therapy has the potential to contribute value by improving outcomes for both carers and patients [25]. Music therapists and art therapists are becoming increasingly available to palliative care teams, and are advancing the diverse and unique clinical services available to effectively meet the holistic needs of patients with serious illnesses and their families [26]. A recent survey identified that a high proportion of UK music therapists who work in end-of-life care (EoLC) also focus their therapeutic practice on family members and care recipients (75.5%) at pre- and post-death bereavement [27]. This can involve dyadic or family-based intervention, in addition to working solely with the carer before and after the patient’s death. This indicates a common clinical need, either identified during music therapist ‘assessment and/or frequently requested by service users, and family members or care recipients in person-centred practice.

However, despite high demand, the role of music therapy in improving outcomes for informal carers remains an under-researched component of EoLC. There is a limited body of existing research (e.g., 28–30) with a recent systematic review [31] only identifying 34 eligible studies on the effectiveness or experience of music therapy with informal carers pre- or post-death bereavement (see study protocol for eligibility criteria https://www.crd.york.ac.uk/prospero/display_record.php?ID=CRD42021244859). The design and methodology of existing research is limited in scope with only two randomised controlled trials (RCTs) and is largely focused on informal carers of individuals with dementia. The existing research is also of variable quality, which can in part be attributed to the methodological challenges of conducting rigorous research in this area. Evaluating arts-based therapeutic approaches within EoLC represents an intersection of two complex areas of work. For example, previous work in this area acknowledged challenges to conducting music therapy RCTs in the field of EoLC [32] including high attrition rates, the need for flexible scheduling of music therapy sessions, and the need for a standardised yet individualised intervention [33].

There is a need for more high-quality music therapy research with a variety of research designs which represents the range of informal carer populations at pre- and post-death bereavement [31]. Patients, families, and healthcare professionals stand to benefit from increased ecological and culturally responsible research about supporting informal carers pre- and post-death bereavement [34], yet in general bereavement support has traditionally received a lack of research funding [35]. With a restricted funding landscape, it is imperative that the focus shift to conducting the ‘best value’ research which is research that addresses the most important questions for improving health services and is conducted to a high standard. A ‘best practice agenda’ involves identifying key priorities to be addressed and how to address methodological challenges. Establishing a ‘best practice agenda’ helps build capacity in research areas which are underdeveloped, a recent example being an agenda for research on group singing, health and wellbeing [36].

## Methods

### Aim

To co-design a best practice agenda for research into music therapy for informal carers of patients pre- and post-death bereavement. This was done using a collective intelligence approach with expert music therapy researchers and clinicians [37].

### Design

A workshop using a World Café [38] approach was held, employing a participatory research methodology that facilitates open and inclusive discussion of views and knowledge with a group of stakeholders. A World Café approach involves creating a cafe-like atmosphere where stakeholders engage in small group conversations around thematic tables. After a set period, stakeholders rotate to different tables, bringing insights and ideas from their previous discussions. This process encourages the exchange of diverse perspectives, fosters open dialogue, and promotes the exploration of complex issues. This study utilised an online World Café approach, an increasingly common format since the global COVID-19 pandemic and when inclusion of international stakeholders is required [39].

### Setting and participants

To facilitate the contribution of international experts a half-day workshop was held via a secure online videoconferencing facility (Zoom) in May 2022. Purposive sampling was used to identify a range of international stakeholders (academics and music therapy clinicians) with expertise in the field of music therapy for supporting informal carers pre- and post-death bereavement (drawn from research networks and published authors in this field).

### Data collection

Fifty potential participants were emailed during March and April 2022 to seek an expression of interest to take part in the World Café workshop. Those who expressed an interest were then emailed a Participant Information Sheet. Two weeks later they were emailed again and asked to complete an online consent form prior to completing a short pre-workshop survey. This short survey (see Supplementary material 1) gathered basic demographics information relating to professional experience, as well as reflections on key priorities and methodological challenges when conducting research with informal carers pre- and post-death bereavement. This information was collated to inform the key themes to be discussed during the 3-hour online workshop.

**Table 1:** Workshop Structure

Initial plenary summarising the evidence to date and important gaps			
Breakout room 1 Priority research areas	Breakout room 2 Theory and ethics	Breakout room 3 Methodological challenges	Breakout room 4 Evaluating Impact
*In four parallel discussion sessions attendees rotate between each room, each building on the discussion and ideas of the previous groups*			
Final large group discussion with summary and consensus building.			

The online workshop was video and audio-recorded (with participants’ consent), and a record was kept of any written responses during sessions for data collection purposes. Findings from the research team’s ongoing systematic review [31] and the data supplied by the stakeholders in the pre-workshop survey were categorised thematically using semantic coding [40, 41]. This informed the thematic focus for the small group discussions and consensus building during the workshop. Ahead of the workshop a timetable, summary of data provided (research priorities and methodological challenges) and guidance on using Zoom was circulated to stakeholders, along with each attendee’s biography, in line with recommendations [41] to ensure all participants understood the context of their work together. Following an initial plenary which summarised the evidence to date and important gaps, the Zoom breakout room feature was used to create discussion “tables” where attendees rotated between four rounds of parallel small group discussions, each lasting 25 min. In face-to-face World Café workshops, the stakeholders would use tablecloths and coloured pens to capture their ideas. As this was online, Padlet software was used to replicate this, with the option to contribute via Padlet without joining the session. A host (one of the research team, two of whom were music therapists, and two academics) remained at each “table” and at the start of each new round provided the new arriving stakeholders with a summary of the previous discussion. This allowed individuals to build on each other’s ideas and experiences. In line with recommendations [38, 41], ideas were ranked in order of importance at the end of each round and each small group discussion involved four stakeholders.

At the end, one final large group discussion involved a summary of discussions and a consensus building activity. This allowed the entire group to make “collective knowledge…visible and actionable” [41]. Stakeholders were given continued access to Padlet for two weeks after the workshop in order to note any further contributions. On reflection, having research team members facilitate the break our rooms may have had the potential to bias the priority setting exercise. However, the discussions were led, and agreed on by the stakeholders to mitigate the research team involvement. See Table 1 for an overview of the workshop Structure.

### Ethics

Formal research ethics approval was obtained from the Engineering and Physical Sciences Faculty Research Ethics Committee in the host university (Reference: EPS 22_109). The study was conducted in accordance with the Declaration of Helsinki [42] and participants completed an informed consent statement prior to completion of the pre-workshop survey.

### Data analysis

All documentation generated prior to the workshop (pre-workshop survey qualitative data) and during the workshop itself (1. notes from breakout room facilitators, 2. Padlet posts) were analysed by TM, and independently validated by KB, AC, LD, ZT, GT and RR using an inductive reflexive thematic approach which involves categorising initial codes into themes and sub-themes [43] to inform priorities and recommendations. A first analysis of priorities for research, methodological challenges, and potential solutions already took place during the workshop through ongoing discussion and refinement of key themes, and through voting on Padlet, so a classical line-by-line coding process was not required [44]. Rigour was increased through the analysis of recordings and transcription (completed by TM), which ensured the context of what was spoken was maintained [44]. Trustworthiness was increased by member checking e.g., participants were asked to provide feedback on the accuracy of the findings to ensure the research team’s interpretation of the data was valid [45].

## Results

Twenty-two of those invited to the workshop (*n* = 50) provided consent to participate in this study and completed the pre-workshop survey (response rate 44%). Fourteen (64%) identified as academics/music therapists, five (23%) as music therapy clinicians, two (9%) as academics, and one (4%) as a PhD student. Respondents had substantial experience in this area ranging from 5 to 37 years, and represented seven countries spanning 10 different time zones, including the United Kingdom, USA, Canada, Australia, Spain, Israel and Serbia.

Seventeen of those who completed the survey joined the online workshop held 25th May 2022, 10.30am-1.30pm GMT. Five were unable to attend but were invited to contribute to the discussion via the online Padlet. Qualitative findings from the survey and workshop are presented under key topics and sub-themes from each of the four online tables (Breakout rooms), resulting in the identification of key priorities and recommendations for a best practice agenda for music therapy research for informal carers of terminally ill patients pre- and post-death bereavement (see Table 3).

### Priority areas for music therapy research to support informal carers of terminally ill patients pre- and post-death bereavement

Breakout room 1 focused on participants’ perceptions of what research areas should be prioritised for informal carers before and during their bereavement. Analysis of their discussions pointed to five key areas presented in the following sections.

#### Priority 1: music therapy across the bereavement continuum

Participants stressed the need to consider music therapy across the bereavement continuum. This includes all of the people who are part of the patient’s family and support system (including children), as well as the patient themselves, and their changing roles and experiences over time. The importance of exploring music therapy in relation to the whole continuum of bereavement resonates with contemporary literature around relationship completion [46, 47]. In music therapy practice, music is often experienced as a “bridge” and a thread of connection not only between the dying person and their carers, but also between people’s changing identities from carers (while their loved one is alive) to bereaved (once they have died). Music therapy’s capacity to foster and maintain this connection is an important feature of “continuing bonds” [48] and a crucial future research area. Participants also highlighted the possibility of exploring “mixed” groups where people come together as both carers and bereaved people to enhance ways of coping.

Music therapy’s role in anticipatory grief and bereavement was also discussed in relation to the mass loss experienced in society during the COVID-19 pandemic. For example, how music therapists may support people at individual, relational, group (e.g. healthcare workers) and wider societal levels with loss and grief associated with the pandemic, especially for those whose grief process was unarticulated or interrupted due to not being able to attend a funeral or take part in collective expressions of grief [49].

#### Priority 2: grief in dementia care.

Participants highlighted dementia-related grief as a specific research area that needs further attention. Globally, increasing numbers of people are caring for family members with dementia. Such people are dealing with ongoing and ever-changing loss, or a series of losses which compound over time, including the ambiguity of grief and loss while their loved one is still alive.

The multiple losses and changes (including loss of memory, autonomy, identity, and selfhood) that the person with dementia and their informal carers will experience [50] call for further exploration around music therapy’s role in this area. Even though it is increasingly a leading cause of death internationally, dementia is not widely considered as a ‘terminal condition’ and many people with dementia sometimes face unnecessary hospital admissions in their dying phase [51]. The need for further evidence to support any specific approach to palliative care in dementia is stressed [52] and this equally applies to pre- and post-death bereavement for their informal carers. Existing evidence and theoretical models regarding grief reactions in dementia carers [53–55] can support music therapy research in this area.

#### Priority 3: psychological wellbeing.

Future research could explore further how music therapy can contribute to carers’ emotional wellbeing and support their ‘transition’ from anticipatory grief to bereavement and coping with loss. Studies beyond music therapy [55] can shed light to emerging foci in this area, including the role of existing social networks and community-based approaches that can support people’s coping with their loss.

Participants also argued that future research should focus on how music therapy can be a viable intervention for people with complicated grief or PGD. This could be explored in relation to early detection and treatment of a complicated grief response and how this could help the prevention of psychiatric and psychosomatic disorders. These areas, including music therapists’ engagement with music as a resource for people’s everyday lives, were also linked to a public health approach to palliative care and its role in the Compassionate Communities initiatives [56]. Adopting a public health approach also aligns with the new HCPC standard for ‘promoting public health and preventing ill-health’ which comes into effect in September 2023 and must be met by all music therapists in the UK [57].

#### Priority 4: group and community work

Participants discussed the role of group music therapy, including community singing groups or hospice community choirs for carers before and during their bereavement. Potential outcome areas included the potential reduction of social isolation, enhancing peer support networks, community development, and mutual understanding, as well as opportunities for challenging myths around grief and loss and opening up conversations that can be considered ‘taboo’.

Participants stressed the need to consider music therapy across the wider experience of bereavement care especially in relation to music therapy’s implementation in different palliative and end-of-life care settings. Participants highlighted the need for exploring how music therapy is implemented - what the potential barriers and facilitators might be to integrating music therapy into different settings, what time frames may be best for each setting (e.g., one-time sessions, workshops, individual or group sessions, time-limited or open-ended), as well as how telehealth might support.

The role of music as a health resource for carers individual psychological and collective wellbeing in everyday life, the potential impact of legacy work in music therapy as well as people’s sense of spirituality in music therapy were all discussed as interconnected issues pertaining to the wider carers’ network [58].

#### Priority 5: equity of care and accessibility

How music therapy can support the anticipatory grief and bereavement experiences of marginalised people and communities is an underdeveloped research area that needs addressing. Such work requires a more in-depth understanding of potential barriers to accessing music therapy at cultural, organisational, and structural levels.

Participants suggested that mapping of service provision can raise awareness of available resources within and beyond specialist palliative care contexts and may enhance the accessibility of appropriate services. Such mappings can include a critical investigation of potential barriers. Funding issues can be part of this, and participants suggested that interdisciplinary collaboration could enhance understanding of health economics and their impact on accessibility and development of music therapy services. In addition to thinking about music therapy, participants discussed the role of music as a resource outside of music therapy and other potential musical care offerings. Questions around accessibility of such resources as well as their relationship to music therapy were highlighted.

### Theory and ethics

Breakout Room 2 explored the philosophies and theories that inform music therapists’ clinical practice and ethical considerations for facilitating research with informal carers of individuals receiving hospice and palliative care. There was a diverse array of thought for each topic, indicating the complexity of each.

#### Theory

Participants identified multiple theoretical positions, some of which were related to specific grief or bereavement theories while others were broader and more conceptual (e.g., person-centredness was the most commonly cited principle). Anticipatory mourning [19], the dual process model of grief [59], and the dementia grief model [60] were specific theories on grief and loss that participants identified as informing their practice. Various attachment theories, including Bowlby’s classic attachment theory [61] and more contemporary frameworks such as interpersonal neurobiology [62], were also cited, as were other psychological theories, such as positive psychology [63].

Multiple theoretical writings specific to music and music therapy were also noted, including community music therapy (“*This lens helps to see all members as equal members of the community*”), music-centred music therapy (“*It is about understanding the unique affordances that music may offer to bereaved carers rather than replicating the goals of other services*”), and resource-oriented music therapy. Research highlighting the power of music included that of Donald Winnicott [64], Tia DeNora [65], Susan Langer [66], John Shepherd and Peter Wicke [67].

Participants also reflected on components of theory they found to be of particular importance and utility. This included participatory action and other types of community engagement, postmodern conceptions of health and wellbeing, and reflexivity to cultural contexts. Music was also noted as a phenomenon to be better understood in action but without the use of reductive lenses that exclusively or primarily frame music as a prescriptive science instead of as a creative, individual experience.

Theory plays an integral role in informing the various parts of clinical practice, including assessment, intervention design, treatment evaluation, and all other forms of clinical decision-making. As discussion among participants in this World Cafe indicated, there is little settled theory specifically for work with informal carers. While there were a number of agreed upon concepts, participants drew from a variety of different sources in discussing those concepts. Relatedly, the music therapy theories cited by participants were not specific to medical settings or any other treatment contexts for that matter; as one participant acknowledged, “*No one size fits all – flexibility is key*.”

Participants in the World Café were broadly in favour of person-centred philosophies that principally located treatment in the therapeutic relationship rather than pre-determined outcomes. Goals and objectives did, however, factor in heavily with participants speaking to the importance of emotional and psychological resilience. This balance reflects comprehensive person-centred theories [68] that understand the support of informal carers to be complex and multidimensional.

#### Ethical issues

Participants consistently spoke to the importance of the carers and also the researchers being taken care of through the process of exploring pre- and post-death bereavement. Comments included:

- *It’s a sensitive area so researchers need to take care of themselves and their respondents.*
- *The timing of this research is important – a focus on bereavement too early or too soon after bereavement may be distressing for caregivers.*
- *Individuals who are experiencing new post- and/or pre-bereavement may not be able to cope with research.*
- *Sometimes bereaved people can say yes to things because they don’t know what else to say, and then find it overwhelming later.*

The issues raised here are largely consistent with the calls for reflexive theories with a participatory action and/or community-engaged focus. Research in this area is with people at particular risk for re-traumatisation, and by extension the researcher is at risk of secondary traumatisation. Approaches to research design and implementation must therefore be attentive to the potential for harm. Participants introduced questions for researchers to be mindful of: *How do we properly involve research participants in a way which allows us to understand their needs and articulate the ethical issues and lived frameworks that affect them? How can we meet the systemic requirements for evidence in a way that does not impose on caregivers in ways that are not helpful to them?*

The ethical implications for research with informal carers are aligned with evolving understandings of the profound impact grief and anticipatory loss can have on carers [53]. Participants collectively asserted the importance of timing when inviting informal carers into research projects: *if the study falls too close or too far away from the death event, researchers can run the risk of retraumatising those carers in distress*. Participatory action research methodologies and social justice paradigms can be instructive in the design, implementation, and reporting of research with informal carers.

### Methodological challenges – improving the quality of the research evidence base

Breakout room 3 focused on participants’ perceptions of methodological challenges to conducting high quality research into music therapy for informal carers before and during their bereavement. The following themes emerged during these discussions:

- defining “quality” research,
- strength in international approach,
- advocacy,
- strength of arts-based research.

#### Defining “quality” research

Defining ‘quality’ research is both a challenge and an opportunity for growth when considering the needs of informal carers and music therapy. Participants discussed the existential issues of defining ‘effectiveness’. Challenges include:

- using guidelines that have not been developed with the complexities of music therapy in mind,
- following the guidelines for research such as RCTs versus the needs of the people who are grieving, and.
- finding support for high quality bids.

Opportunities include challenging the definition of “gold standard” and remaining a humanity centred healthcare profession. As one participant stated, “*Gold standard RCT’s are challenging but we can challenge that.*”

Conducting the traditional gold standard research via RCT [69] to evidence effectiveness in music therapy and palliative care research is notoriously difficult for all of the reasons cited in the literature [25, 70] and also discussed by the participants in the World Café workshop. Challenges include small sample sizes, high attrition, and blinding of participants to name but a few. However, pilot and feasibility studies can help gather the evidence required to guide the key methodological decisions required to optimise an RCT’s success [71] when this is deemed the best approach to answer the research question.

Thankfully there is growing interest and collective efforts to ensure that palliative care research [70], and the arts and humanities [72], are not disadvantaged when it comes to conducting high quality research. The MORECare [70] statement was developed to provide evidence-based guidance for those wishing to conduct high quality palliative care research. Recognising the inherent challenges of conducting RCTs in this area, they provided alternative best practice designs. These included a waitlist design to avoid participants missing out on a potentially beneficial intervention delivered as part of an RCT and use of a cluster randomised trial to minimise the risk of contamination and aid recruitment; all the while acknowledging that these designs are not without their own challenges, including requiring a large sample size to ensure adequate power to detect change.

The Medical Research Council have also recently updated their guidance for developing and evaluating complex interventions [72] which reflects the concerns and priorities put forward by the World Café participants. The old guidance was based on the outdated paradigm that prioritised the ‘does it work’ effectiveness question which failed to address whether the intervention would be cost-effective, implementable, and scalable in real world practice. Echoing what participants recommended, the new guidance emphasises the importance of using a mixed method, theory-based approach (such as realist review/evaluation) to evaluate if a complex intervention, such as music therapy for supporting informal carers of terminally ill patients pre- and post-death bereavement, is effective, for whom, and in what circumstances. This is much more useful for those having to make decisions around commissioning of services as it provides evidence for the feasibility of an intervention, recommendations for implementation, cost effectiveness, scalability and transferability across different contexts.

Alongside the practical limitations inherent in RCTs, participants also discussed the undercurrent of deeper epistemological challenges of attempting to determine effectiveness of a complex intervention that needs to be flexible and individualised to match client needs using an evidence-based practice approach [73]. One way to address this complexity and meet the design requirements for an RCT of person-centred disciplines such as music therapy is through embedding a realist evaluation, and via manualisation of therapies to ensure treatment fidelity, which was successfully achieved in a recent pilot and feasibility RCT of a music therapy intervention for hospice inpatients [74].

#### Strength in international approach

While the profession of music therapy is small compared to other healthcare professions such as nursing, there can be strengths in a small community. Engaging music therapists in an international study is possible. A mixed methods international study was discussed by the participants to draw on the strengths of a larger music therapy community. That this is achievable is also evidenced by the recent successful HOMESIDE RCT, of an online home-based music intervention for Family Carers of People Living with Dementia [75]. This three-arm RCT involved five countries including Australia, Germany, UK, Norway, and Poland, and recruited 432 patient/carer dyads.

#### Advocacy

Advocacy reflects the need to contribute to the literature on carers from a music therapy perspective, to educate others on music therapy, and to challenge the accepted definition of evidence, causation and generalisability [76]. Music therapists have unique knowledge related to the profession, to health, grief, and to the people they work with. Music therapists often advocate for the profession and clients. Formalising this skill through a research agenda and lens will further the needs of the profession and better help carers access music therapy.

Advocacy is a necessary skill for a profession such as music therapy which is largely underused and underfunded, both in services and in research. Music therapists throughout their careers find themselves having to advocate, either for services or research projects to be set up, or to enhance the understanding of others involved within services or research projects. The aim of this is to help the profession grow and enhance opportunities for service users to receive the support they need. Developing the research and therefore the evidence base for the profession helps to support the arguments made to advance a particular position or perspective and build credibility. Formally considering the potential advocacy impact of any research project in this area during its conception, development and design will help to ensure that the research meets this need and is of the best possible benefit to the profession, and therefore to its service users. Research topics should be selected to be the most of benefit to the development of the profession, and researchers should be prepared to perhaps move beyond the immediate research environment and use their skills and expertise to support advocacy for the profession, proudly sharing their research projects.

#### Strength of arts-based research

The strength of an arts-based approach to research reflects an emerging worldview that builds on the expertise of music therapists. As one participant stated, “*As part of arts-based approaches to research, creative output and dissemination can engage people in what we do*.” Sharing the actual music from music therapy sessions may help non-music therapists understand the evidence of music therapy sessions, the phenomena of informal carers’ bereavement experiences, the unique contexts of different situations, and ultimately build upon non-music therapists’ relationship to music to increase understanding of music therapy.

Music therapists have a unique opportunity to use the creative outputs that their work may have (with consent) to support the outcomes ‘on paper’ of their research and help people to understand the impact found. This is an additional channel that can help portray the complexities of both informal carers’ bereavement experiences and the impact of music therapy on them. As the creative activity lies at the heart of the therapy process it can also be central to the research itself. This supports the participatory research approach that participants described as being necessary.

While the pre-agreed focus of this discussion room was on methodological challenges, the outcomes of the discussion seemed to flip the perspective, focussing more on strengths that can be built on in order to address challenges that may be met – the need to define what is required to carry out ‘quality’ research and how this does not align neatly with the complexities of the music therapy profession; the strengths in an international approach (can help address any challenges); the need for ongoing advocacy; and the strength of arts-based approaches.

### Evaluating the impact of music therapy pre- and post-death bereavement/optimal follow-up assessment period

Participants discussed benefits and challenges of various outcome methods during breakout room 4 and put forward suggestions for ways that music therapy outcomes could be best measured in future research. Three subthemes were developed for this theme:

- Challenges of Measuring Impact;
- Preferred Ways to Measure Impact; and.
- Suggested Follow-Up Timeframe.

#### Challenges of measuring impact

Participants discussed challenges of capturing the impact of music therapy in research. There was a general consensus amongst participants that standardised measures were *‘not sensitive to music therapy interventions’*, and do not always capture the full experience of music therapy participants. Qualitative data was described as important due to a close alignment with the person-centred therapeutic approaches that the participants used in their practice. However, participants also recognised that qualitative research does not always align with the priorities of funding bodies. One participant noted that while validated outcome measures are important, qualitative data is needed to explain results that might not be captured by quantitative data measured:

I think music therapy affects more “proximal” outcomes that are “on the way” towards things like “reduced burden” or reduction on anxiety and depression. So, we need the qualitative data to contextualise/explain this.

Participants also highlighted some limitations of standardised measures. The length of time for caregivers to complete some validated measures was highlighted as a potential burden, as well as a factor that could contribute to biased data:

I am mindful of stories from research participants who do not enter authentic data through fatigue and frustration with longer repetitive scales.

Despite the wide range of outcome measures identified, discussions during the World Café workshop focussed around the difficulty of choosing validated outcome measures that adequately capture the breadth and depth of what music therapy aims to do. Core outcome sets (COS) are one way of directing researchers to the most clinically relevant outcomes to include in RCTs [77]. A recent review by Harrop et al. [78] identified ‘ability to cope with grief’ and ‘quality of life and mental wellbeing’ as COS for bereavement support, with 21 dimensions that relate to these outcomes also described. The core outcomes identified in Harrop et al.’s [78] review align with three of the outcome domains suggested by participants in the World Café workshops (Coping with Grief, Quality of Life and Psychological Wellbeing), while the 21 dimensions also encapsulate other domains (including Physical Health and Social Support), and both align with outcomes identified in the systematic review aligned with this project [31]. Notably, the COS identified by Harrop et al. [78] originated from qualitative literature and group discussions, and the authors suggest that past inconclusive evidence in the bereavement support literature may be due to incongruence between the aims and methods of interventions and outcomes measured [23, 79]. This also reflects the discussions from the World Café workshops, in which participants highlighted the discrepancies between the evidence desired by funding bodies and the realities of their practice. The importance of qualitative data was emphasised throughout the workshops and is supported by the literature [23, 78, 80]. This confirms the importance of including the perspectives of people with lived experience, in addition to relevant quantitative data to best measure and understand the impact of music therapy for bereaved populations. However, further research is needed to determine which standardised measures are best, and/or to develop new tools to best measure the COS for bereavement.

#### Preferred ways to measure impact

Participants described a range of outcome measures and data collection methods to evaluate the impact of music therapy **(**see Table 2 for an overview of the outcome measures previously used by participants). Capturing participants’ perspectives was seen to be of chief importance due to the complex nature of experience and alignment with person-centred approaches. In addition to qualitative interviews and participant diaries, capturing *“…‘hallway’ feedback that is given in response/ following session”* was also suggested as a potential way to understand the more immediate impact of music therapy.

For quantitative outcome measures, seven outcome domains were raised, while the need for new music therapy specific and/or *“bespoke”* measures was also discussed. Health Economics approaches (such as collecting data on participants’ medical costs) was suggested as an alternative way of capturing data around caregiver health without requiring caregivers to complete lengthy questionnaires.

**Table 2:** Outcome measures used by participants

Outcome Domain	Recommended Outcome Measures
Coping with Grief	- Hogan Grief Reaction Checklist [81] - Grief-specific questionnaires
Aspects of Caregiver Experience	- Positive Aspects of Caregiving [82] - Caregiver Resilience Scale [83] - Zarit Caregiver Burden [84] - Revised Scale for Caregiver Self-Efficacy [85] - NPI Subscale (Caregiver Distress) [86]
Quality of Life	- EQ-5D-5 L (Health Related Quality of Life) [87] - Quality of Life questionnaire [88] - Assessment of Quality of Life – 6 Dimension (AqoL-6D) [89]
Social Support	- Multidimensional Scale of Perceived Social Support (MPSS) [90]
Physical Outcomes	- The MOS 36-item short-form health survey (SF-36) [91] - The Patient Health Questionnaire (PHQ-9) [92] - Health Economics (analysis of medical costs)
Psychological Outcomes	- Beck Depression Scale [93]
Music Therapy Evaluation	- The Impact Areas Questionnaire (IAQ): (A Music Therapy Service Evaluation Tool) [94] - Bespoke Evaluation Process

The pre-workshop survey responses and workshop discussions revealed a diverse range of standardised measures used by participants to assess music therapy’s impact on grief specific outcomes, quality of life (QoL) outcomes, social support outcomes, physical health outcomes, psychological outcomes, and bespoke outcome measures developed by the music therapists (see Table 2). The majority of these outcomes aligned with outcomes identified in a previous systematic review of the music therapy and bereavement literature [31]. However, the inclusion of physical outcomes is notable, as the review did not identify any music therapy studies that used these outcomes. The physical impact of bereavement has been well documented [95, 96], and correlations between bereavement and increased cost to health care systems have also been noted [97]. Therefore, we recommend the inclusion of physical outcomes in future research.

Some participants also described bespoke outcome measures that they had used in their own practice, although no validated examples were provided. One music therapy-derived tool, the Impact Areas Questionnaire [94] was suggested as an evaluation tool that could capture the perspectives of service users and those who support them, staff and organisations. Although this tool is designed to evaluate clinical services as a whole, rather than a participant’s individual outcomes, it may be a particularly useful tool in helping communicate the impact of music therapy to funding bodies.

#### Suggested follow-up timeframe

The need for both immediate (i.e., straight after a session) and longer-term follow-up was noted in order to fully understand the potential benefits of music therapy. While suggested timeframes for longer-term follow-up varied from six weeks to 12 months, three months was repeatedly noted, as *“often around the time that immediate supports (extra help from family/friends etc.) might drop off”.* Mixed method approaches to follow-up (i.e., qualitative interviews and self-report questionnaires) were recommended, however, as quoted above, one participant acknowledged that lengthy interviews at follow-up may not provide *“authentic data [due to] fatigue and frustration”*.

Considerations around the timing of follow-up assessment was also captured in the pre-workshop survey and workshop. As expected, there was consensus that follow-up assessment should be conducted immediately or as soon as possible after the final music therapy session to capture any short-term benefit. Recommendations for longer term follow-up ranged from 6 weeks to 12 months, with three months viewed as the optimal time to assess any long-term benefit of music therapy, in line with the time around which family and/or friend support has dropped off.

Determining the longer-term effects of healthcare interventions for chronic conditions (such as pre- and post-death bereavement) is usually achieved via an RCT which includes long-term follow-up. However, challenges such as loss to follow-up, and treatment switching (confounding of outcomes from other therapies post-intervention completion) must be considered [98]. If including long-term follow-up in trial designs, these challenges should be anticipated and planned for, so that the data required for statistical analyses to account for these challenges is available [98].

### Overall recommendations

The recommendations made above have been summarised in the following Table 3, aligned to the phases of development of a research project.

**Table 3:** Recommendations aligned to research design phases

	Consider the following:
Overall approach to study topic	The full bereavement continuum, including transition from anticipatory grief to bereavement and loss.
	Potential for long-term effects of grief
	The wider ecology of bereavement care
	Potential issues of diversity and equality
	Potential issues of equity and accessibility of care
Research design	Interdisciplinary and international collaboration
	Mixed method, theory-based approach (such as realist review/evaluation embedded in an RCT) to evaluate if a complex intervention, such as music therapy for supporting informal carers of terminally ill patients pre- and post-death bereavement, is effective, for whom, and in what circumstances
	Involvement of carers as research collaborators throughout the research project (not just as participants)
	Theoretical perspective, bearing in mind wide possible range
	Prioritise due care for participants and also researchers
Approach to intervention design	Potential for public health approaches
	Relationship-oriented and person-centred practice
Methodology / Outcomes measures	Mixed methods design
	Include physical outcomes
	Use Core Outcome Set for evaluation of bereavement interventions
	Consider burden on participants when selecting outcomes measures
Data collection	Consider arts-based approaches
	Innovative approaches to data collection
	Inclusion of health economics data
	Timeframe for data collection – immediately post-intervention and 3-month follow-up

## Discussion

Our aim in this work was to develop a best practice agenda for music therapy research to support informal carers of terminally ill patients pre- and post-death bereavement using a World Café approach [38]. Findings indicate that the top priority overall was evidencing the impact or benefit of music therapy across the bereavement continuum. From a summary of the results, we can extrapolate the requirements for doing this. Our expert stakeholders agreed that we need to use mixed methods, as though robust trial data is needed to provide ‘effectiveness’ data, we need qualitative data to understand why, how, and in what circumstances music therapy is more likely to benefit service users.

Community music therapy initiatives – including intergenerational musical care practices for death education [99, 100] as well as broader health promoting and public health palliative and bereavement care initiatives [34, 101] can provide fertile ground for future research, offering opportunities for mixed participant groups including carers and bereaved relatives, as well as people with incurable illnesses. In this context, ecology points to an understanding of the different palliative and end-of-life care contexts where music therapy can be offered. Different layers of the care ecology, from micro to macro levels [102], can offer a lens to explore not only the nuanced musicking encounters between people, but also music therapy’s role beyond ‘session time’ and its wider positioning within the organisational life of a care environment. This includes music therapy’s role in promoting connections between different people who are part of the wider bereavement experience and fostering a culture where music can be used as a health resource in people’s everyday life. Existing practices and evidence regarding the role of legacy work [103] as well as people’s sense of spirituality in music therapy [99, 104] can inform future research of this wider ecology of care.

We also need to use the existing core bereavement outcome set [78], so that music therapy interventions can demonstrate value against the wider bereavement evidence base. The important timepoints to consider for evidencing benefit are believed to be immediately after the music therapy intervention has completed and again 3 months later. And finally, we need to measure cost-effectiveness as this is what commissioners of services need to know in terms of decision making, namely whether or not investing in music therapy services will save the NHS money through improved carer outcomes.

Finally, theory plays an integral role in informing the various parts of clinical practice, including assessment, intervention design, treatment evaluation, and all other forms of clinical decision-making. The choice of theory should be guided by individual need, but person-centred theories were the favoured approach within the world café, perhaps because they position treatment in the therapeutic relationship rather than on pre-determined outcomes which can be complex and multidimensional when supporting informal carers through bereavement.

Given the known complexities of carrying out research both into the impact of music therapy treatment and within the context of palliative and end-of-life care, it is important that researchers can be supported and facilitated to carry out research that is as high quality as possible. A best practice agenda refers to a set of easy-to-understand guidelines and principles that are the most effective and efficient methods for achieving a particular goal or objective and for obtaining the best possible outcomes. By being aware of and following a best practice framework or approach, researchers can improve the outcomes and quality of their projects. Best practice is often developed using collective wisdom, knowledge and experience, and the World Café approach enabled us to do this, bringing together a number of international experts in this field and combining their input.

The World Café workshop, including information gathered in the pre-workshop phase, during the workshop event itself, and the Padlet platform, provided rich sources of data which effectively gathered the input, knowledge, and experience of all those who participated. A range of national and international perspectives were highlighted across the four key themes resulting in several key recommendations to form the best practice agenda for research in this area.

### Limitations

This study has several potential limitations. The international stakeholders invited to take part in the World Café event were primarily drawn from authors of the published research in this area (identified in the authors’ systematic review). Although this is an appropriate approach to identify individuals who have significant experience of music therapy research and can speak to methodological challenges and solutions, other disciplines outside of music therapy were not represented. This may have impacted on the areas identified for future research. It also should be noted that experts identified from the existing research were identified from studies published in English. There are likely to be expert stakeholders in this area that the research team are unaware of, and we acknowledge that those included were from high-income countries. Although we don’t feel this is likely to impact the generalisability of the methodological recommendations, it may limit the applicability of priority areas for low-and-middle income countries. Lastly, informal carers were not involved in the consensus process. The inclusion and exclusion criteria for stakeholder invitations were discussed with our expert carer advisory group who advised against including carers in this phase of the project, given the focus on research methods.

A further potential limitation is the involvement of some co-authors (KB, AC, LD, ZT, GT and RR) in the World Café workshop which may have biased the findings. However, it is common for this type of exercise to include the expert stakeholders as authors [74]. Stakeholder co-authors were only included after data collection and helped to shape the paper based on their expertise in this area.

## Conclusions

Establishing a ‘best practice agenda’ helps to build capacity in research areas which are underdeveloped, and best practice for music therapy research does not appear to have been formally outlined in the literature. Many of the considerations identified could apply more widely to other areas of music therapy and also palliative care research rather than necessarily being specific to informal carers pre- and post-bereavement.

### Electronic supplementary material

Below is the link to the electronic supplementary material.

**Supplementary Material 1:** Short pre-workshop survey

## Data Availability

The datasets used and/or analysed during the current study are available from the corresponding author on reasonable request.

## Abbreviations

COS: Core Outcome Set
EoLC: End-of-Life Care
EoL: End-of-Life
MPSS: Multidimensional Scale of Perceived Social Support
PGD: Prolonged Grief Disorder
