# Supplementary material for: Developing a best-practice agenda for music therapy research to support informal carers of terminally ill patients pre- and post-death bereavement: a world café approach
Source: BMC Palliat Care. 2024 Feb 7;23:33. doi: 10.1186/s12904-024-01369-8 (PMC10851575; doi:10.1186/s12904-024-01369-8)
Supplement: Supplementary file 1 — Supplementary Material 1: Short pre-workshop survey [file 12904_2024_1369_MOESM1_ESM.docx]

**Online World Café Event Survey**

**Developing a best-practice agenda for music therapy research to support informal carers of terminally ill patients pre- and post-death bereavement: a World Café approach**

1. **How would you describe your gender?**

Man

Woman

Non-binary

Prefer not to say

1. **How would you describe your ethnicity?**

White

Mixed or Multiple ethnic groups

Asian or Asian British/American/European

Black, African, Caribbean or Black British/American/European

Other ethnic group

1. **What is your age band?**

18-30

31-40

41-50

51-60

61+

1. **What is your profession/area of expertise?**

Music therapist (clinical practice only)

Academic/Music therapist

Academic (teaching/research only)

PhD student

Other [please specify]

1. How many years' experience do you have in conducting/involvement in music therapy research with informal carers pre- and post-bereavement?
2. Have you published any research in this area?

If yes, how many publications?

1. What country do you reside in?
2. Could you please provide a short biography (maximum 100 words) below to share with other delegates at the workshop? Please include your affiliation, position, research interest(s), and citations for recent relevant publications (if you have any)

**PRIORITY SETTING**

1. What research area(s) should we be focusing on with informal carers pre- and post-bereavement and why is it important?

**THEORY**

1. What theoretical frameworks do you use in your music therapy research with informal carers pre- and post-bereavement and why?

**ETHICAL ISSUES**

1. What ethical issues do you think researchers in this area need to be aware of, and how can they be addressed?

**RESEARCH DESIGN ISSUES**

1. What research design do you feel is most appropriate for developing the evidence base in this area? What are the advantages and disadvantages of this design?
2. If you have been involved in conducting randomised-controlled trials (RCTs) in this area, what are the key challenges of using this design in music therapy research with informal carers pre- and post-bereavement? (e.g. randomisation, control group, blinding)
3. What outcome measure(s) have you used in your music therapy research with informal carers pre- and post-bereavement - advantages/disadvantages?
4. What are the optimal follow-up assessment point(s) in order to demonstrate longer-term benefit from music therapy with informal carers pre- and post-bereavement?

**PUBLISHING STRATEGIES**

1. Which journals do you try to publish in and why?
2. Which journals (music and non/music) have you had most success publishing with?

**FUNDING STRATEGIES**

1. What funder(s) do you apply to for your music therapy research with informal carers pre- and post-bereavement?
2. Which funder(s) have you had most success with?
